# Analysis of population-level determinants of legionellosis: spatial and geovisual methods for enhancing classification of high-risk areas

**DOI:** 10.1186/s12942-017-0118-4

**Published:** 2017-12-02

**Authors:** Jessie A. Gleason, Kathleen M. Ross, Rebecca D. Greeley

**Affiliations:** 1 Environmental and Occupational Health Surveillance Program, New Jersey Department of Health, Trenton, NJ USA; 2 Communicable Disease Service, New Jersey Department of Health, Trenton, NJ USA; 3 Council of State and Territorial Epidemiologists Applied Epidemiology Fellowship, Atlanta, GA USA

**Keywords:** Legionellosis, Legionnaires’ disease, Cluster analysis, GIS, SaTScan™, Ecological study

## Abstract

**Background:**

Although the incidence of legionellosis throughout North America and Europe continues to increase, public health investigations have not been able to identify a common exposure in most cases. Over 80% of cases are sporadic with no known source. To better understand the role of the macro-environment in legionellosis risk, a retrospective ecological study assessed associations between population-level measures of demographic, socioeconomic, and environmental factors and high-risk areas.

**Methods:**

Geographic variability and clustering of legionellosis was explored in our study setting using the following methods: unadjusted and standardized incidence rate and SaTScan™ cluster detection methods using default scanning window of 1 and 50% as well as a reliability score methodology. Methods for classification of “high-risk” census tracts (areas roughly equivalent to a neighborhood with average population of 4000) for each of the spatial methods are presented. Univariate and multivariate logistic regression analyses were used to estimate associations with sociodemographic factors: population ≥ 65 years of age, non-white race, Hispanic ethnicity, poverty, less than or some high school education; housing factors: housing vacant, renter-occupied, and built pre-1950 and pre-1970; and whether drinking water is groundwater or surface water source.

**Results:**

Census tracts with high percentages of poverty, Hispanic population, and non-white population were more likely to be classified as high-risk for legionellosis versus a low-risk census tract. Vacant housing, renter-occupied housing, and homes built pre-1970 were also important positively associated risk factors. Drinking water source was not found to be associated with legionellosis incidence.

**Discussion:**

Census tract level demographic, socioeconomic, and environmental characteristics are important risk factors of legionellosis and add to our understanding of the macro-environment for legionellosis occurrence. Our findings can be used by public health professionals to target disease prevention communication to vulnerable populations. Future studies are needed to explore the exact mechanisms by which these risk factors may influence legionellosis clustering.

## Background

Since the organism was first recognized during an outbreak in 1976, *Legionella pneumophila* has been identified as a relatively common cause of pneumonia in community and healthcare settings and has continued to garner attention globally [1]. Approximately 5000 cases of Legionnaires’ disease are reported each year in the United States [2]; however, the actual number of hospitalized cases is estimated to be between 8000 and 18,000 [3] and the number of reported cases have been steadily increasing in the United States, Europe and Canada [4–7]. Although the first case of Legionnaire’s Disease was recognized over 40 years ago, it remains a disease of emerging concern particularly in developed countries with more surveillance [1].

Legionellosis refers to two syndromes caused by bacteria of the genus *Legionella* including: (1) Legionnaires’ disease, which is the more severe form of the infection and associated with pneumonia; and (2) Pontiac fever, which is milder and not associated with pneumonia [8]. Transmission to humans occurs through inhalation of aerosolized water that is contaminated with the bacteria. Legionellosis is not transmitted between individuals or by swallowing contaminated water; however, aspiration is also an important mode of disease transmission. *Legionella* species are common worldwide in the natural environment, including rivers, streams, and lakes and in artificial water environments and can survive in a range of environmental conditions, but grows best in warm temperatures between 32 and 42 °C (90–108  F) [1].

The risk of contracting legionellosis is multifactorial, including factors which promote the proliferation of *Legionella* in the environment, host susceptibility factors, and exposure to aerosols. Known host susceptibility factors associated with the occurrence of legionellosis include older age (> 50 years), male sex, history of smoking, chronic lung disease, and poor immune function [9]. Outbreak investigations have identified the following environmental and exposure to aerosol risk factors: history of travel, residence in a healthcare facility, and proximity to water systems including: domestic water services (tanks, showers, faucets, stagnant warm pipes); cooling towers and evaporative condensers; whirlpool spas; respiratory therapy devices; vegetable misters; ice machines; and decorative fountains systems [1, 10, 11]. However, outbreaks of legionellosis account for only a minority of cases. Up to 80% of legionellosis cases are sporadic and public health investigations have not been able identify a common source, although these cases are not randomly distributed in time or space [12–14].

Research of population-level factors may be a useful complement to individual-level factor research which is the primary stream of research into causes and factors of legionellosis [15]. Some research has identified potential common population-level exposures including meteorological factors such as temperature, humidity, and water pressure [11, 16, 17], wastewater treatment plants [18], certain public water system characteristics [19], and other natural matrices and man-made water systems [11]; yet further investigation into population-level factors is needed to provide a better understanding of the complex nature of legionellosis. Although other studies of area-based predictors have assessed associations with infectious disease including giardiasis [20], salmonellosis [21], and campylobacteriosis [22], similar studies of legionellosis are limited.

Previous research has demonstrated methods for detection of small areas of excess incidence of Legionnaires’ disease [23] as well as cluster detection as a prospective surveillance tool [24]. However, there is a paucity of ecological studies comparing legionellosis rates across geographic subsets (e.g., county, census tract) to estimate the impact of environmental and sociodemographic health disparities [25]. Incidence analyses are useful tools to describe and compare disease rates across geographic subsets. There is no steadfast rule to categorize incidence rates to identify areas of high occurrence and choice of cut point may lead to an over- or under- estimate of high occurrence areas. Use of spatial cluster analyses to identify areas of high occurrence can supplement findings from incidence rate analysis.

Studies which focus on spatial disease clustering may be successful at identifying macro-level factors, such as socioeconomic, demographic, and environmental factors, which contribute to the increase in disease [26]. SaTScan™ is a commonly used cluster detection software which calculates a spatial scan statistic by gradually scanning a window across space, noting the number of observed and expected observations inside the window at each location [27]. SaTScan™ users can adjust the size of the cluster scanning window (i.e., percent of the total population at risk); however, results can be misleading if the size of the cluster scanning window is made arbitrarily since a small scanning window can produce unstable clusters whereas a large window can overestimate the cluster area [28]. Researchers need to determine which model (e.g., maximum window size) best represents the true underlying clusters and how to quantitatively assess the fit of a model [29].

As increases in the incidence of legionellosis are observed, further investigation of population-level determinants of legionellosis is needed to expand our understanding of the complex mechanism of this disease. An ecological analysis was performed to estimate the association between legionellosis risk and the following population-level risk factors: age, race, ethnicity, poverty, education, housing factors including vacant, renter-occupied, and housing age, as well as drinking water source. To best classify census tracts at “high-risk” for legionellosis we explored the following spatial variability methods: (1) unadjusted and standardized incidence ratios across census tracts; (2) cluster detection with minimum and maximum window scanning and a reliability score method for classification of homogenous, reliable legionellosis clusters. The identification of geographic areas with high burden of disease and subsequently evaluating population-level determinants could allow for targeted public health interventions.

## Methods

### Study population and design

Our study setting was the state of New Jersey, the most densely populated of the 50 United States (U.S.), with a population of just under 9 million people. New Jersey is adjacent to New York City, New York the most populous city in the U.S., and Philadelphia, Pennsylvania the 5th most populous city. This statewide study setting provides a template for states/regions/countries to assess the role of population-level factors and legionellosis and not restrict research to urban city centers. Census tracts, areas roughly equivalent to a neighborhood with an average population of 4000, were used as geographic unit of measure. Census tracts were developed and used by the U.S. Bureau of Census to collect, tabulate and present results of the decennial censuses. There are 2000 populated census tracts in New Jersey. Although U.S. census blocks, which contain about 1000 people, may provide finer scale geospatial analysis, data at that granular level is more limited and information for all factors of interest is not available.

A clinically compatible legionellosis case is classified as confirmed if it meets at least one of the confirmatory laboratory criteria: (1) culture isolation of any *Legionella* organism from respiratory secretions, lung tissue, pleural fluid, or other normally sterile fluid; (2) detection of *L. pneumophila* serogroup 1 antigen in urine [30]; or seroconversion of fourfold or greater rise in specific serum antibody titer to *L. pneumophila* serogroup 1. In accordance with communicable disease reporting regulations set by the New Jersey Department of Health (NJDOH), healthcare providers must report diagnosed cases of legionellosis within 24 h of laboratory confirmation to the local health department where the case resides. Local health departments are responsible for initiating a public health investigation and subsequently reporting the case to the State health department through NJDOH’s Communicable Disease Reporting and Surveillance System (CDRSS).

Confirmed legionellosis cases from 2003 to 2013 were extracted from CDRSS. Cases that were out-of-state during the entire incubation period (2–10 days prior to illness onset) were excluded from the study. Cases were also excluded if the CDRSS record did not contain age at onset, gender, date of symptom onset, or address. Legionellosis cases were geocoded to census tract based on their street address using ArcGIS version 10.2. Each census tract was assigned the aggregated count of legionellosis cases, the total 2010 U.S. Census population, and geographic coordinates of the census tract centroid for utilization in SaTScan™ software.

Census tract level demographic and socioeconomic variables of interest were obtained from the 2010 U.S. Census including percentages of total population ≥ 65 years of age, non-white race, Hispanic ethnicity, below poverty, less than or some high school education. Housing variables included percentages of housing units which are vacant, renter-occupied, and built pre-1950 and pre-1970. Both pre-1950 and pre-1970 housing were explored in univariate analyses allowing for a simple comparison of effect estimate magnitude, but only pre-1950 was included in multivariate analyses. Each continuous exposure variable was categorized using the lower and upper quartiles rounded to the nearest 5% as cutoffs. The cutoffs, with distributions, for each variable can be found in Table 2. Additionally, each census tract population-weighted centroid was spatially joined to the public water system in which it fell. The primary drinking water source of each public water system was classified into ground water, surface water, and unknown.

### Spatial methods

The following methods were used to explore geographic variability and clustering of legionellosis and to identify associations with population-level factors: (1) unadjusted and standardized incidence rate methods and (2) cluster detection using both 1 and 50% window size sensitivity as well as a reliability score method to minimize window size sensitivity. Unadjusted legionellosis incidence rates were calculated for the 10-year study period for each census tract by dividing the number of confirmed legionellosis cases per census tract by total census tract population. Age- and sex-adjusted standardized incidence rates were calculated for the 10-year study period for each census tract using direct standardization. New Jersey’s age- and sex-category population estimates available from the 2010 U.S. Census were used as the standard population. A choropleth map of the unadjusted and age- and sex-adjusted incidence rates was created using ArcGIS. Adjusted and unadjusted incidence rates were categorized into five intervals (0, > 0 and ≤ 2; > 2 and ≤ 4; > 4 and ≤ 6; and > 6) for visualization.

### Cluster analysis

Discrete Poisson models were used to identify retrospective, purely spatial clusters of legionellosis, using SaTScan™ software [31]. Standard clusters were defined as statistically significant clusters identified with SaTScan™ using the maximum scanning window size (50% of the population at risk) and a minimum window size of 1%. To mitigate SaTScan™ window size sensitivity and maximize the detection of stable legionellosis clusters, we calculated individual reliability scores for each census tract utilizing a methodology introduced by Chen et al. [28]. SaTScan™ was run 50 times adjusting the size of the cluster scanning window (1–50%) by 1% with each run. Each SaTScan™ run used a maximum likelihood function that identified the most likely cluster and secondary statistically significant clusters. *P* values obtained through Monte Carlo hypothesis testing using 999 replications and relative risk values were assigned to each census tract.

Using the output from the 50 SaTScan™ runs, reliability scores were calculated for each census tract as described in Chen et al. [28]. Reliability score is calculated by R_i_ = C_i_/S, where R_i_ is the reliability score for location i, C_i_ is the number of scans for which that location i is within a significant cluster, and S is the total number of SaTScan™ runs; and is defined as the likelihood that a census tract is reported within a significant cluster among systematically varying cluster scanning window sizes. Reliability scores can range from zero to one; zero indicates that the census tract was not identified within any statistically significant clusters in any SaTScan™ scans and one means that the census tract was identified within a statistically significant cluster in every SaTScan™ run.

Public health analyses routinely use choropleth maps to display incidence and cluster data, however Roth et al. [32] describe methods to improve the visualization of reliable, homogenous, and high-risk clusters. By utilizing this proposed methodology, reliability scores and relative risk values were concurrently illustrated with bivariate choropleth map using ArcGIS. This technique used a two-dimensional color and transparency legend (Fig. 3).

### Statistical analysis

Univariate logistic regression modeling was used to explore associations between population-level factors and three types of legionellosis occurrence (i.e., census tract identified through conventional incidence rate; default cluster methods; and reliable, high-risk cluster methods) with demographic, socioeconomic, and environmental risk factors. Census tracts were categorized as high-risk if the incidence rate was > 2 cases per 100,000 persons per year (incidence rate during 10-year study period was divided by 10 to estimate annual rate) or if the census tract was located within a statistically significant cluster detected by the default cluster detection methods. Census tracts with a reliability score ≥ 0.5 and a RR ≥ 2 were categorized as high-risk for cluster detection using the reliability score method. These classification criteria are presented in Table 1.

**Table 1:** Methods use to assess spatial variability of legionellosis, classification criteria for high-risk census tracts, and corresponding number of census tracts, cases, and estimated population captured by each method

Method of spatial variability	“High-risk” classification	# of census tracts identified as “high-risk”	# of cases residing in “high-risk” census tract	Estimated population within “high-risk” census tract
Unadjusted IR	IR ≥ 2 per 100,000	744	1407	3,126,058
Standardized IR	IR ≥ 2 per 100,000	724	1376	3,151,260
1% cluster detection	Detected within a cluster	93	243	327,824
50% cluster detection	Detected within a cluster	259	412	981,983
Reliable cluster detection	RS ≥ 0.5 and a RR ≥ 2	136	397	507,694

*IR* incidence rate, *RS* reliability score, *RR* relative risk

Multivariate analyses explored associations of demographic, socioeconomic, and environmental risk factors of reliable, high-risk cluster census tracts compared to low-risk census tracts (RS = 0 and RR < 2) while simultaneously controlling for the other factors. Census tracts which were associated with a cluster but did not meet the criteria of a reliable, high-risk cluster were not included in the multivariate analyses. Pairwise correlations between each continuous exposure variable, pre-categorization, was examined. Collinearity diagnostics were assessed with an a priori cutoff of 30 for the condition indices (CI). Stepwise logistic regression was used to create a model that required a significance level of 0.05 to allow a variable into the model and a significance level of 0.05 to remain in the model. Forward and backward model selection resulted in the same final model; while the full model had the lowest AIC value. Results are reported as adjusted odds ratios with 95% confidence intervals.

## Results

### Descriptive data

From 2003 to 2013, 1634 reported legionellosis cases in New Jersey met the study’s inclusion criteria. Pairwise Pearson correlation coefficients between continuous predictor factors identified only weak and very weak correlations (r < 0.39) except for a moderate positive correlation between education and non-white race (r = 0.42), poverty and Hispanic ethnicity (r = 0.49), and a strong a correlation between renter-occupied and some high school education (r = 0.61) and renter-occupied and non-white race (r = 0.61).

### Incidence rate

**Fig. 1:**
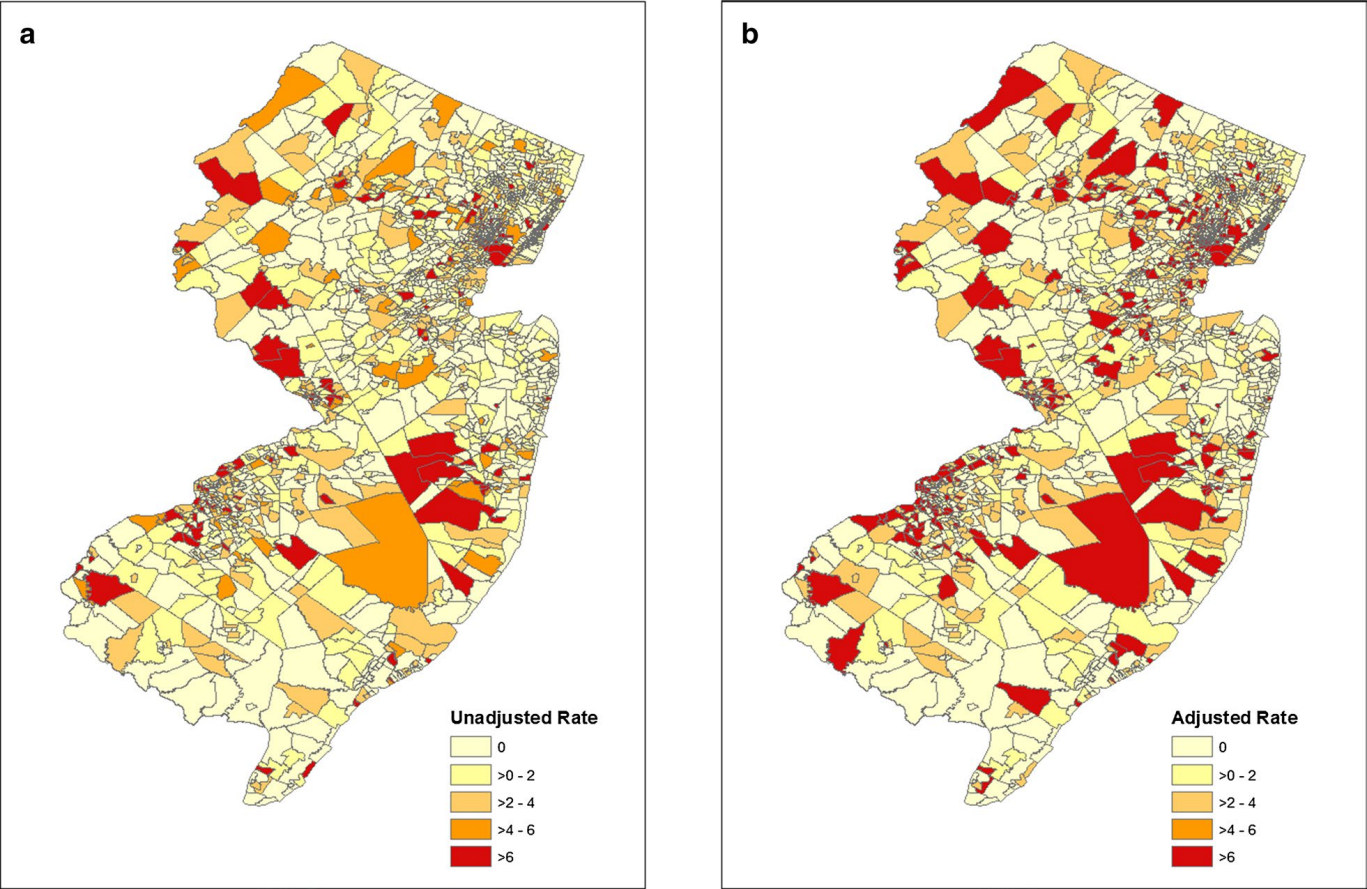
Unadjusted and age and sex-adjusted legionellosis incidence rates by census tract in New Jersey, USA, 2003–2013

Although incidence rate methods included the largest number of cases, these methods also have a correspondingly large number of census tracts and population size which will likely result in dampened effect estimates when modeled (Table 1). Cluster analyses had fewer census tracts identified, with the reliability score method appearing to maximize the number of cases included against number of census tracts as compared to the default 1 and 50% cluster methods. Among the 2000 populated census tracts in New Jersey, 969 (49%) tracts had at least one confirmed case of legionellosis between 2003 and 2013 (range 1–10 cases). The average 10-year unadjusted incidence rate was 19.5 cases per 100,000 persons (range 0–264) and the average 10-year adjusted incidence rate was 20.8 cases per 100,000 persons (range 0–1694) (Fig. 1a, b).

### Cluster detection

**Fig. 2:**
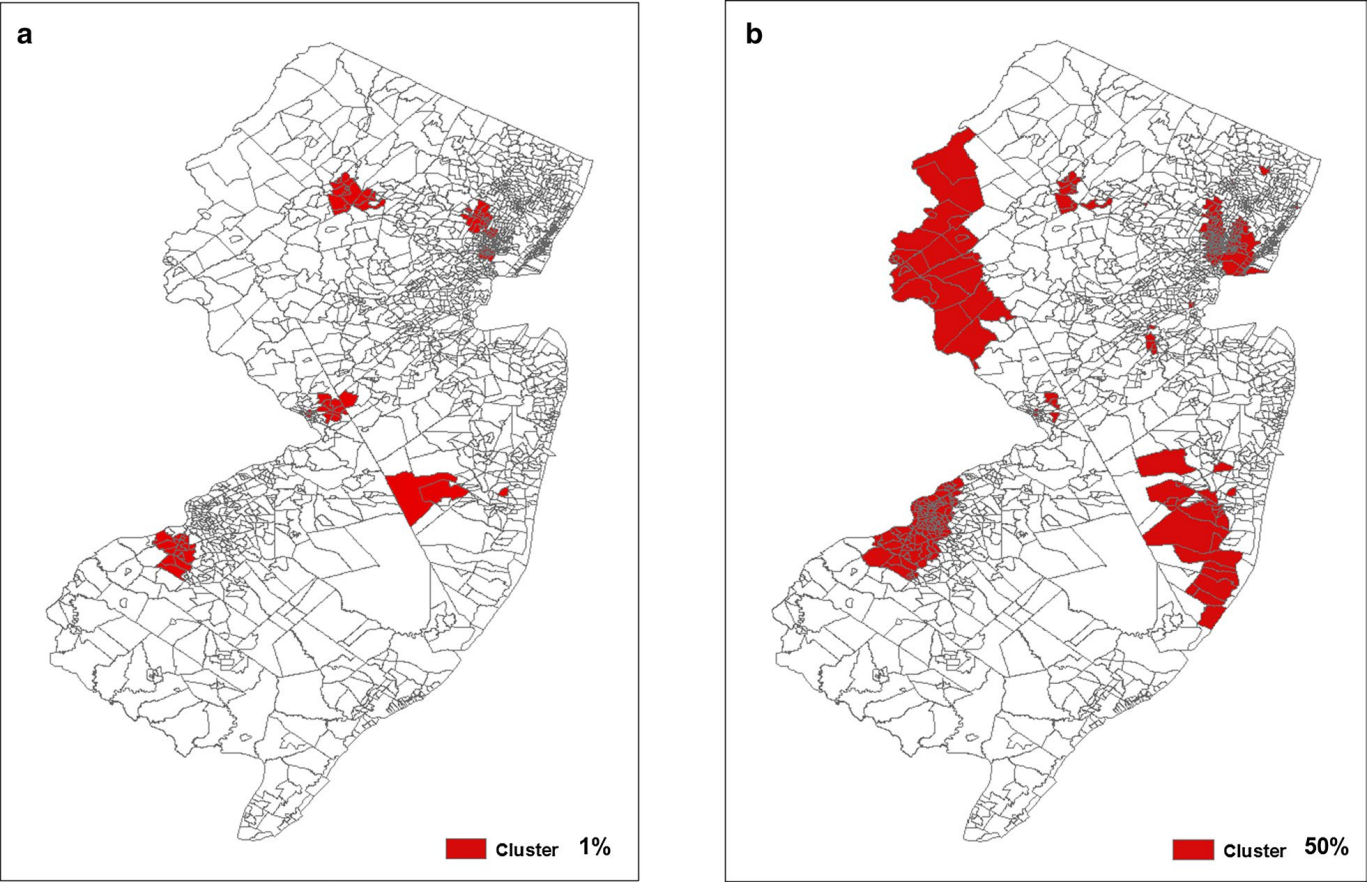
Statistically significant legionellosis clusters detected using SaTScan™ software with census tract as geographic unit with 1 and 50% of the population at risk in New Jersey, USA, 2003–2013

**Fig. 3:**
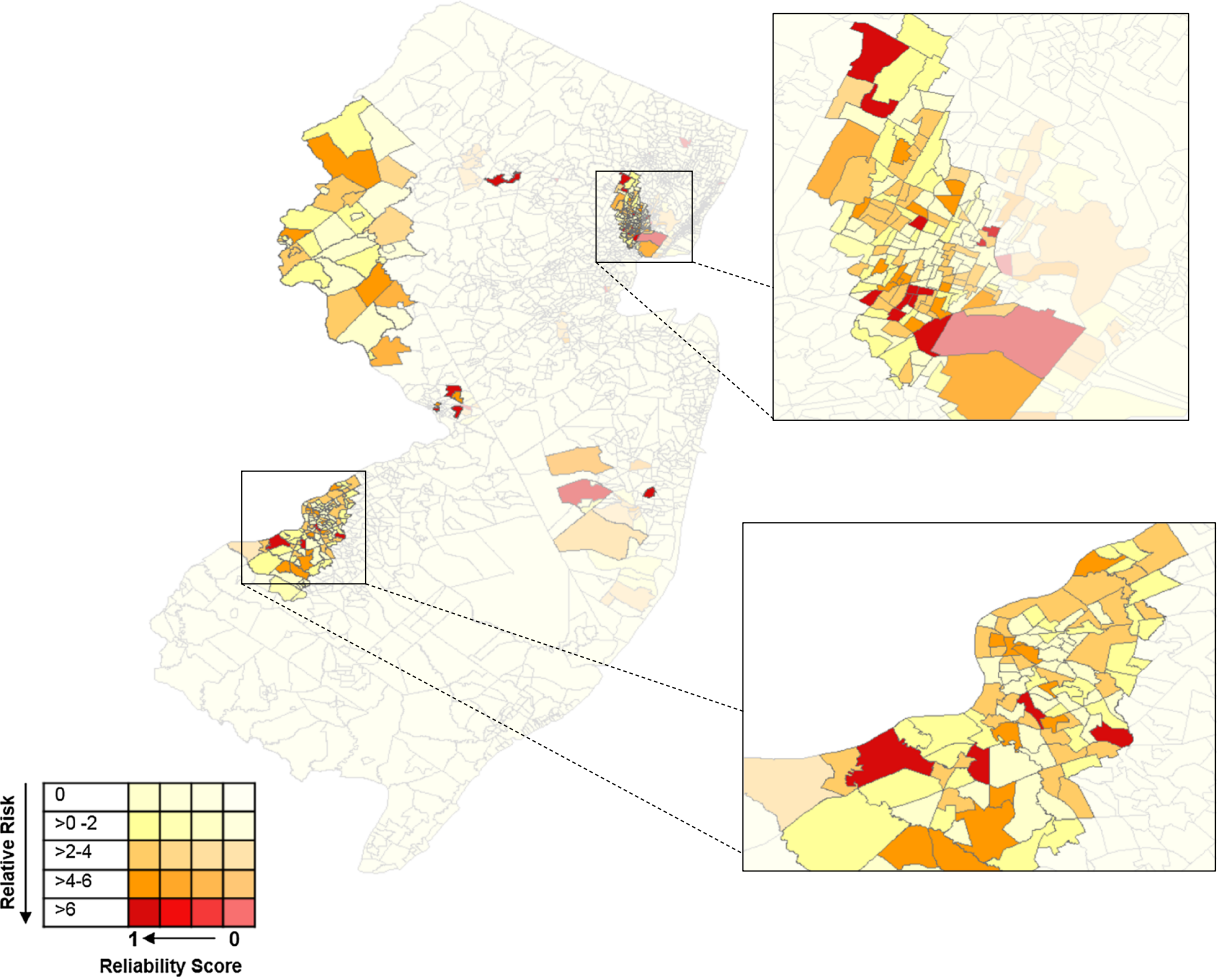
Reliable, high-risk legionellosis clusters detected utilizing reliability score methodology and SaTScan™ software with census tract as geographic unit, in New Jersey, USA, 2003–2013. The variable was stratified into 0.1 increments and each increment was assigned its own data layer. Census tracts with a reliability score of 0 were presented at 80% transparency and with each reliability score category a 20% transparency deduction was made until the census tracts with the highest relatability scores would be fully opaque. Within each transparency layer, relative risk was categorized into five groups, each assigned a distinct color. The highest relative risk category was assigned a bold, dark red. With each decreasing relative risk category, the color scheme became more muted such that the lowest category was assigned a very pale yellow/nude color

SaTScan™ cluster detection was performed with a minimum and maximum size scanning window of 1 and 50% (Fig. 2a, b). The 1% scanning window resulted in 11 statistically significant clusters comprised of 93 census tracts, while the scanning window of 50% resulted in six statistically significant clusters comprised of 259 census tracts. Alternatively, the methodology proposed by Chen et al. [28] identified 322 (16%) census tracts as belonging within at least one statistically significant cluster, of which 136 census tracts met the high-risk definition (RS ≥ 0.5 and RR ≥ 2) (Fig. 3).

### Statistical analyses

Unadjusted odds ratios and 95% confidence intervals of associations with population-level risk factors highlights differences among spatial methods of legionellosis occurrence (i.e., conventional incidence rate; default cluster census tracts; reliable, high-risk cluster census tracts) (Table 2). Census tracts classified as high-risk through the incidence rate method were more likely to be categorized into the highest non-white population category (OR 1.67; 95% CI 1.28, 2.17); percentage of poverty category (OR 1.49; 95% CI 1.17, 1.90); lower education level; and housing units that are renter- occupied or built pre-1950 or pre-1970 as compared with census tracts with lower rates of legionellosis. Comparatively, census tracts classified as high-risk through cluster analysis, both default and reliability score methods, found similar patterns of positive associations with population-level factors as those from incidence analysis methods, but effect estimates were much stronger for all factors explored. For example, census tracts classified as high-risk for incidence analysis were approximately 1.5 times more likely to be categorized into high poverty than lower risk census tracts compared with 3.8 times more likely that cluster-based high-risk census tracts would be categorized as high poverty. Cluster methods also found statistically significant associations with older age and vacant housing. Only minor, non-significant, differences were noted between findings from default cluster analyses and reliability score methods. The final multivariate logistic regression model found that census tracts with the highest percentages of older age (OR 0.38; 95% CI 0.22, 0.67); Hispanic populations (OR 0.10; 95% CI 0.05, 0.19); poverty (OR 7.21; 95% CI 4.04, 12.86); and housing units built pre-1950 (OR 5.69; 95% CI 2.82, 11.50) were positively statistically significantly associated with reliable, high-risk legionellosis cluster areas (Table 3). It is important to note that once other factors were controlled for, Hispanic ethnicity had a negative, statistically significant association.

**Table 2:** Unadjusted odds ratios and 95% confidence intervals of macro-level demographic and environmental factors and increased odds of legionellosis occurrence

	Incidence rate^a^		Standard cluster^b^		Reliable, high-risk cluster^c^	
	OR	95% CI	OR	95% CI	OR	95% CI
% ≥ 65 years of age						
< 10	–		–		–	
≥ 10 to < 15	1.10	0.88–1.37	*0.60*	*0.44*–*0.81*	*0.45*	*0.30*–*0.67*
≥ 15	0.84	0.66–1.07	*0.49*	*0.35*–*0.69*	*0.33*	*0.20*–*0.53*
% non-white race						
< 10	–		–		–	
≥ 10 to < 50	1.00	0.79–1.26	0.81	0.57–1.17	0.89	0.54–1.47
≥ 50	*1.67*	*1.28*–*2.17*	*2.67*	*1.86*–*3.83*	*3.07*	*1.87*–*5.02*
% hispanic ethnicity						
< 5	–		–		–	
≥ 5 to < 20	1.19	0.96–1.48	*0.66*	*0.49*–*0.88*	1.05	0.70–1.58
≥ 20	1.08	0.84–1.39	*0.47*	*0.32*–*0.67*	0.78	0.47–1.28
% poverty						
< 5	–		–		–	
≥ 5 to < 15	*1.34*	*1.09*–*1.65*	*1.87*	*1.34*–*2.62*	1.40	0.86–2.25
≥ 15	*1.49*	*1.17*–*1.90*	*3.78*	*2.68*–*5.32*	*5.04*	*3.24*–*7.84*
% less or some high school						
< 5	–		–		–	
≥ 5 to < 10	1.24	0.99–1.55	1.22	0.85–1.75	1.52	0.92–2.48
≥ 10	*1.30*	*1.04*–*1.62*	*2.35*	*1.70*–*3.25*	*2.70*	*1.72*–*4.25*
% homes built pre-1950						
< 5	–		–		–	
≥ 5 to < 20	*1.45*	*1.17*–*1.80*	*4.93*	*3.02*–*8.06*	*4.08*	*2.24*–*7.43*
≥ 20	*1.48*	*1.13*–*1.92*	*9.93*	*5.97*–*16.50*	*6.58*	*3.50*–*12.35*
% homes built pre-1970						
< 15	–		–		–	
≥ 15 to < 35	*1.58*	*1.25*–*2.00*	*2.22*	*1.45*–*3.40*	*1.72*	*1.02*–*2.91*
≥ 35	*1.78*	*1.32*–*2.42*	*5.33*	*3.35*–*8.49*	*3.94*	*2.21*–*7.04*
% renter occupied						
< 10	–		–		–	
≥ 10 to < 55	1.06	0.84–1.34	1.41	0.96–2.09	1.50	0.86–2.61
≥ 55	*1.40*	*1.08*–*1.82*	*2.97*	*2.00*–*4.42*	*4.02*	*2.31*–*7.00*
% vacant housing						
< 5	–		–		–	
≥ 5 to < 10	0.85	0.69–1.04	*1.42*	*1.04*–*1.95*	*1.81*	*1.16*–*2.82*
≥ 10	1.22	0.96–1.56	*2.54*	*1.82*–*3.55*	*3.58*	*2.27*–*5.66*
Primary water source^d^						
Groundwater	–		–		–	
Surface water	0.77	0.60–1.00	0.80	0.56–1.15	0.90	0.56–1.45

Missing values were excluded from analysis

Values in italics are statistically significantly at a significance level of 5%

Ecological study design at the census tract level

^a^Age- and sex-adjusted incidence greater or equal to 2 cases per 100,000 persons

^b^Significant cluster with 50% of population at risk, relative risk greater or equal to 2 cases per 100,000 persons

^c^Exposed census tracts defined as RR > 2 and reliability score > 0.5

^d^Population-weighted centroids of census tracts were joined with public water system polygons

**Table 3:** Adjusted odds ratios and 95% confidence intervals of macro-level demographic and environmental factors and increased odds of legionellosis occurrence

	Model A^a^		Model B^b^	
	OR	95% CI	OR	95% CI
% ≥ 65 years of age				
< 10	–		–	
≥ 10 to < 15	*0.62*	*0.40*–*0.97*	0.65	0.41–1.03
≥ 15	*0.38*	*0.22*–*0.67*	*0.38*	*0.21*–*0.70*
% non-white race				
< 10	–		–	
≥ 10 to < 50	0.85	0.49–1.48	0.82	0.45–1.51
≥ 50	1.68	0.92–3.07	1.27	0.61–2.65
% hispanic ethnicity				
< 5	–		–	
≥ 5 to < 20	*0.46*	*0.28*–*0.74*	*0.46*	*0.28*–*0.75*
≥ 20	*0.10*	*0.05*–*0.19*	*0.10*	*0.05*–*0.21*
% poverty				
< 5	–		–	
≥ 5 to < 15	1.52	0.91–2.52	1.39	0.82–2.33
≥ 15	*7.21*	*4.04*–*12.86*	*6.26*	*3.46*–*11.34*
% less or some high school				
< 5	–		–	
≥ 5 to < 10			1.37	0.78–2.39
≥ 10			1.25	0.67–2.32
% homes built pre-1950				
< 5	–		–	
≥ 5 to < 20	*4.63*	*2.43*–*8.82*	*4.52*	*2.37*–*8.65*
≥ 20	*5.69*	*2.82*–*11.50*	*5.38*	*2.66*–*10.91*
% renter occupied				
< 10	–		–	
≥ 10 to < 55			0.99	0.51–1.95
≥ 55			1.28	0.54–3.02
% vacant housing				
< 5	–		–	
≥ 5 to < 10			1.19	0.70–2.03
≥ 10			1.49	0.81–2.74
Primary water source^c^				
Groundwater	–		–	
Surface water			0.70	0.41–1.22

Missing values were excluded from analysis

Values in italics are statistically significantly at a significance level of 5%

Ecological study design at the census tract level

^a^Multivariate stepwise logistic regression model

^b^Full multivariate logistic regression model

^c^Population-weighted centroids of census tracts were joined with public water system polygons

## Discussion

Our study assessed the relationship between population-level factors and areas of higher legionellosis occurrence. Census tracts classified as high-risk for legionellosis were more likely to have a high percentage of non-white population, poverty; low education level; and high percentage of housing units that are renter- occupied or built pre-1950 or pre-1970. Following adjustment of covariates, high percentage of older age, poverty, and housing units built pre-1950 were positively associated while high percentage of Hispanic populations was negatively associated. Cluster detection methods for classifying high-risk census tracts were preferred from the incidence rate method.

### Spatial methods

Little guidance exists for determining cut-off selection for incidence rates to classify high-risk census tracts. In 2009, the crude U.S. national incidence rate for legionellosis was 1.15 per 100,000 persons, the age-adjusted was 1.08 per 100,000 persons [33]. Therefore, a priori, study investigators selected an IR > 2 (or IR > 20 for 10-year rate) as a “high-risk” cut-off; which appears to have over-selected for high-risk census tract, with almost a third of the state’s population included in a high-risk census tract. In general, our findings suggest that the potential over-selection led to limited ability of both univariate and multivariate models to either detect significant associations or resulted in more moderated effect estimates as compared with the cluster-based methodologies. Population-level analyses are important for contributing to our understanding of the macro-level determinants of legionellosis, and these analyses require accurate identification of at-risk areas.

Comparatively, results from incidence analyses and cluster detection methods supported each other; yet cluster detection methods, both default and reliability score methods, detected much stronger associations for all factors explored. However, we found that results did not meaningfully differ based on cluster detection method and default window scanning sizes are effective in estimating associations. Public health authorities can apply Chen’s methodology for retrospective surveillance to identify homogenous, reliable, high-risk disease clusters for prioritizing targeted prevention outreach.

### Study results and literature

As the percent of persons aged ≥ 65 years increases from 10 to 14% to greater than 15%, the negative association with legionellosis clustering becomes stronger. Since people 50 years of age or older are known to be at increased risk for acquiring legionellosis [1], further research is required to determine why older age was a protective factor in this study. After controlling for other factors, census tracts with a ≥ 5 to < 20% Hispanic population and ≥ 20% Hispanic population were 54 and 90% times less likely, respectively, to be classified as high-risk for legionellosis clustering. As with older age, Hispanic ethnicity was less likely to be associated with increased incidence or clustering or legionellosis. A recently published study that assessed race/ethnicity and Legionnaires’ Disease incidence in New York (2002–2011) had similar findings; the average incidence per year for non-Hispanic blacks was significantly higher than that for Hispanics [10]. Census tracts with more than 50% non-white populations were significantly more likely to be associated with legionellosis incidence and clustering, although, this effect was not seen while controlling for other factors, such as poverty. Associations with legionellosis and race were therefore largely driven by confounding from poverty, which was anticipated by a moderate correlation between the two continuous variables, and as suggested by previous research [15].

In both multivariate models, poverty level remained the strongest risk factor for legionellosis. The positive association between poverty level and legionellosis clustering strengthened as other factors, such as ethnicity and race were controlled for. The data show a distinct gradient in risk as the percentage of poverty level increases. Overall, incidence of Legionnaires’ disease in the city of New York increased 230% from 2002 to 2009 and followed a socioeconomic gradient, with highest incidence occurring in the highest poverty areas [10, 26]. Among patients with community-acquired cases, the probability of working in transportation, repair, protective services, cleaning, or construction was significantly higher for those with Legionnaires’ disease than for the general working population. Although socioeconomic status may be linked with risk factors for legionellosis (e.g., smoking, travel, underlying illness), a direct association between socioeconomic status and the risk of legionellosis has not been identified [34]. Factors related to socioeconomic status, recognized as an important determinant of certain chronic conditions, has not been extensively evaluated for its role in the incidence of infectious diseases. Incomplete information in case reports regarding individual level socioeconomic factors in many U.S. disease surveillance systems reduces the usefulness of surveillance data for these determinants. To our knowledge, no prior studies using group level data investigated the relationship between socioeconomic factors and legionellosis.

In our study, census tracts with higher proportions of rented (vs. owned) housing units were strongly associated with legionellosis incidence and clustering, although, this effect was not seen while controlling for other factors, such as poverty and pre-1950 housing. According to the American Housing Survey, rental units are more likely to be structurally inadequate than owner occupied units [35]. Given that older housing is also more likely to be inadequate, more than 13% of rentals built before 1960 have some structural deficiencies [36]. Older housing stock was also strongly associated with legionellosis clustering and areas of high occurrence. These effects appear to have a dose–response such that effect estimates were stronger for census tracts from the moderate to highest category of older housing for both pre-1950 or pre-1970, and were more elevate for older pre-1950 versus pre-1970 housing stock. Older housing stock issues may be two-fold at both the individual and area-based level. Not only are older homes at risk of deteriorating plumbing systems leading to poor water quality, census tracts with older housing stock may also have an aging community water infrastructure providing homes with poorer water quality. Even when controlling for poverty and source of water, pre-1950 housing was strongly associated with legionellosis occurrence. Transmission of legionellosis has been reported to occur in private homes, yet as most outbreak investigations are focused on public buildings, outbreaks are more easily associated with large public buildings as compared with private residences [25]. There is a need for investigations to address how individuals acquire *Legionella* from their home, especially as these residential homes and infrastructures continue to age [37–39].

Public water distribution systems have been shown to play a role in the transmission of *Legionella* including potential contamination of the plumbing systems of buildings [40, 41]. Since surface water is exposed to the environment, it was hypothesized that *Legionella* may be found at higher concentrations in census tracts served by these systems. Therefore, census tracts served by surface water sources may be at an increased risk of legionellosis occurrence compared with groundwater sources. However, no statistically significant associations with drinking water source water were found.

Pairwise correlations between population-level factors were not as strongly correlated as we predicted, since macro-environment factors are often predictive of one another (e.g. poverty and race, older housing stock, education levels). Area-based measures may be inherently collinear which may make multivariate analyses problematic. Condition indices were well below the cutoff of 30 and stepwise regression appears to have removed the more highly correlated factors. Future studies are needed to explore the exact mechanisms by which these risk factors may influence disease.

### Limitations

Our study has inherent limitations based on its ecological study design. The associations identified between legionellosis and risk factors were measured at the census tract level and cannot be used to make individual inferences—this would result in biased interpretations known as ecological fallacy. Our cluster detection methods are limited to use of home address only; which could potentially result in exposure misclassification. This study did not have the capability to detect work-related or travel-related clusters. A similar study conducted in New York City found that certain occupations might be associated with increased risk for community-acquired legionellosis [10]. We recommend that in the future, public health investigations collect work addresses during patient interviews and medical record reviews to enable prospective cluster detection surveillance to identify work-related disease clusters. Due to limited data availability at the census tract level additional risk factors such as co-morbidities and smoking were not explored and future research should include these important factors.

## Conclusion

Along with well-established risk factors (e.g., older age, sex, immunocompromised), emerging variables such as socioeconomic and environmental factors may be significant indicators for legionellosis risk at the census tract level. The findings from this study are particularly important because they create an opportunity for public health authorities to better target communication for disease prevention. While geographic patterns alone are insufficient to conclude that these factors are causally related to the risk of legionellosis, these methods can be regarded as a first step approach in the evaluation of census tract level factors and the risk of legionellosis.
